# Natural dyes versus lysochrome dyes in cheiloscopy: A comparative evaluation

**DOI:** 10.4103/0974-2948.71051

**Published:** 2010

**Authors:** Narendra Nath Singh, V R Brave, Shally Khanna

**Affiliations:** *Department of Oral Pathology and Microbiology, Kothiwal Dental College and Research Centre, Moradabad, U.P, India*

**Keywords:** Cheiloscopy, lip print, lipstick, sudan black, vermilion, indigo dye

## Abstract

Cheiloscopy is the study of lip prints. Lip prints are genotypically determined and are unique, and stable. At the site of crime, lip prints can be either visible or latent. To develop lip prints for study purpose various chemicals such as lysochrome dyes, fluorescent dyes, etc. are available which are very expensive. Vermilion (Sindoor used by married Indian women) and indigo dye (fabric whitener) are readily available, naturally derived, and cost-effective reagents available in India.

**Objective:**

To compare the efficacy of sudan black, vermilion, and indigo in developing visible and latent lip prints made on bone china cup, satin fabric, and cotton fabric.

**Materials and Methods:**

Out of 45 Volunteers 15 lip prints were made on bone China cup 15 lip prints on Satin fabric and 15 on Cotton fabric. Sudan black, vermilion and indigo were applied on visible and latent lip prints and graded as good (+,+), fair (+), and poor (-) and statistically evaluated.

**Results:**

The vermilion and indigo dye gives comparable results to that of sudan black for developing visible and latent lip prints.

## Introduction

Cheiloscopy (from the Greek words cheilos lip, skopein to observe), is the name given to the lip print studies.[1]

Tsuchihashi named the wrinkles and grooves visible on the lips as ‘sulci labiorum rubrorum’. The imprint produced by these grooves is termed ‘lip print’, the examination of which is referred to as ‘cheiloscopy’.[2]

In a crime scene investigation, lip prints can link a subject to a specific location if found on clothes or other objects, such as glasses, cups or even cigarette butts.[1] Lip prints in the form of lipstick smears are frequently encountered in forensic science laboratories as one of the most important forms of transfer evidence.[3]

Lipsticks are complex substances, which have in their constitution, oils such as modified castor oil, waxes, organic inks, and inorganic pigments for colour.[1,3] Traditional lipsticks produce a lip print that can easily be studied i.e. visible lip print. But, lip prints obtained with persistent or long lasting lipsticks which does not leave a visible smear due to their minimal oil content and those obtained from non-lipstick-coated lips are considered as latent prints.[4]

In criminal identification, latent print evidence is often considered the key in solving a crime.[1] Also latent print can be used as a DNA source because epithelial cells could be retrieved from the print, so as to double its identifying value.[5,6]

It has been documented that lip prints either visible or latent could be developed successfully for study purpose using lysochrome dyes, such as sudan black, sudan III, oil red O, and fluorescent dyes such as Nile red.[5]

Development of lip prints can be made using several substances, such as aluminium powder, silver metallic powder, silver nitrate powder, plumb carbonate powder, fat black aniline dye, or cobalt oxide. All lip prints contain lipids which make their development possible by using lysochrome dyes, such as sudan III, oil red O, and sudan black. The use of fluorescent agents is required when the colour of the developer and the colour of the surface on which the lip print lies are the same, or when the lip print is an old brand.[1]

Mercedes Alvarez Segui *et al*. in 2000 found aluminium and magnetic powders to be effective for developing latent lip prints.[7]

Ana Castello *et al*. in 2002 studied long lasting lipstick prints on porous surfaces using lysochromes and concluded that lysochromes are a highly useful group of compounds for locating and developing recent as well as older latent lip prints.[4]

Ana Castello *et al*. in 2005 and later in 2006 showed that Nile Red was a very effective reagent to develop old latent lip prints on porous surfaces and when the print was deposited on multicoloured or dark surfaces.[8,5]

Esperanza Navarro *et al*. in 2006 showed that sudan III, oil red O, and sudan black are effective for obtaining recent invisible lipstick-contaminated lip mark on corpse’s skin.[6]

Vermilion, commonly known as Sindoor is used by married Hindu women along the hair parting line to signify that they are married. It is an opaque orangish red pigment, originally derived from mineral Cinnabar and is chemically mercuric sulphide.[9]

Indigo dye is a fabric whitener that is naturally derived from plant of genus Indigofera which are native to the tropics and is chemically Indigotin.[10] Both vermilion and indigo are very cost-effective and readily available as compared to sudan black. So, the following study was conducted on students of Kothiwal Dental College and Research Centre, Moradabad, to compare the efficacy of sudan black, vermilion, and indigo in developing visible and latent lip prints obtained on bone china cup, satin fabric, and cotton fabric.

## Materials and Methods

### Materials

1. Traditional lipstick (Lakme, Hindustan Lever Ltd., Mumbai)
2. Long lasting lipstick (Lakme, Hindustan Lever Ltd., Mumbai)
3. Bone china cup
4. White satin fabric
5. White cotton fabric
6. Camel hair brush

### Reagents

1. Sudan black (National Chemicals, Vadodara)
2. Vermilion (Clarion, Kolkata cosmetics, Kolkata)
3. Indigo dye (Robin Blue, Reckitt Benckiser (India) Ltd, Jammu)

### Methods

#### Collection of sample

Lipstick was applied on the vermilion zone of forty five volunteers and after one minute fixation, lip prints were made by fifteen volunteers on cup, fifteen volunteers on white satin fabric and by remaining fifteen volunteers on white cotton fabric. Six lip prints were obtained by one volunteer: three with traditional lipstick which leaves visible prints and remaining three with long lasting lipstick which leaves latent prints.

#### Development with sudan black, vermilion, and indigo

Immediately after collecting visible and latent lip prints, using a camel hair brush, a small quantity of sudan black dye in powder form was applied on visible and latent prints. Application of dye was continued until the print became clearly visible for the study. Same procedure was followed using vermilion and indigo.

Visible and latent lip prints developed with sudan black, vermilion, and indigo were then compared for clarity of lip grooves.

## Results

**Figure 1:**
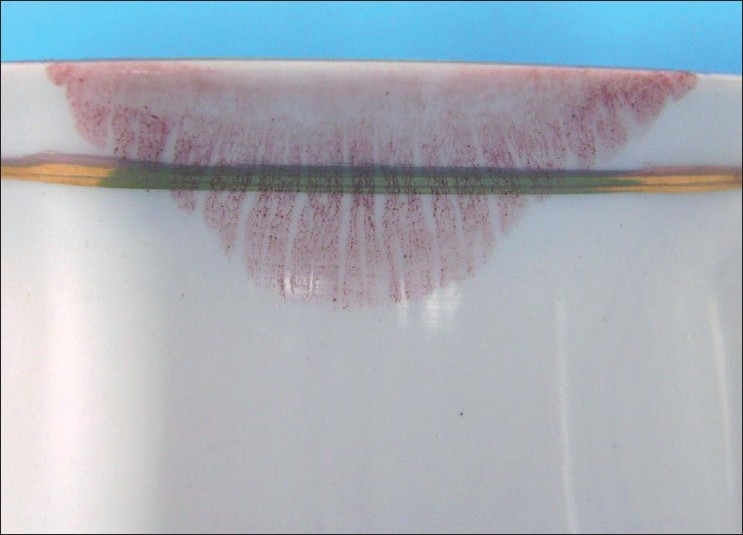
Visible lip prints

**Figure 2:**
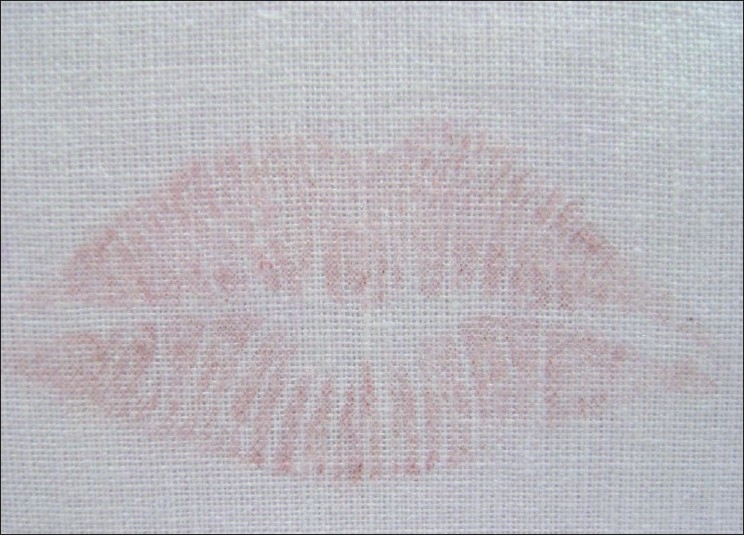
Visible lip prints

According to the aforementioned method, lip prints were obtained, developed immediately, and images were grabbed hence forth:

Visible Lip Prints [Figures 1 and 2]

**Figure 3:**
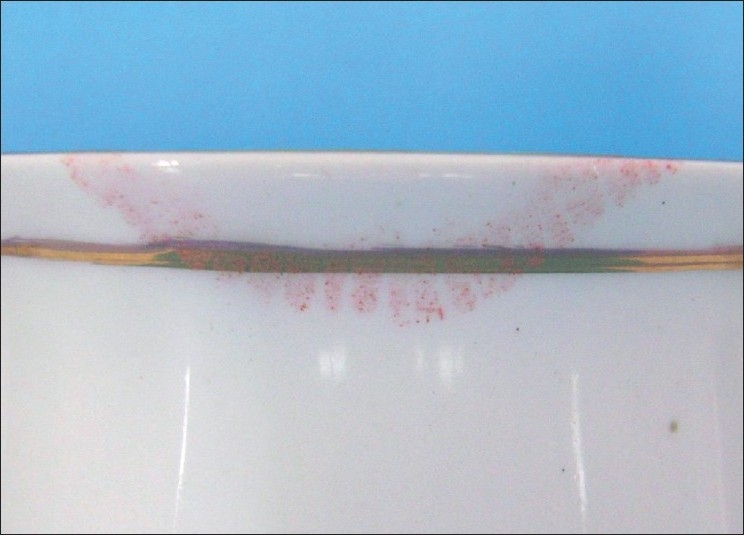
Latent lip prints

**Figure 4:**
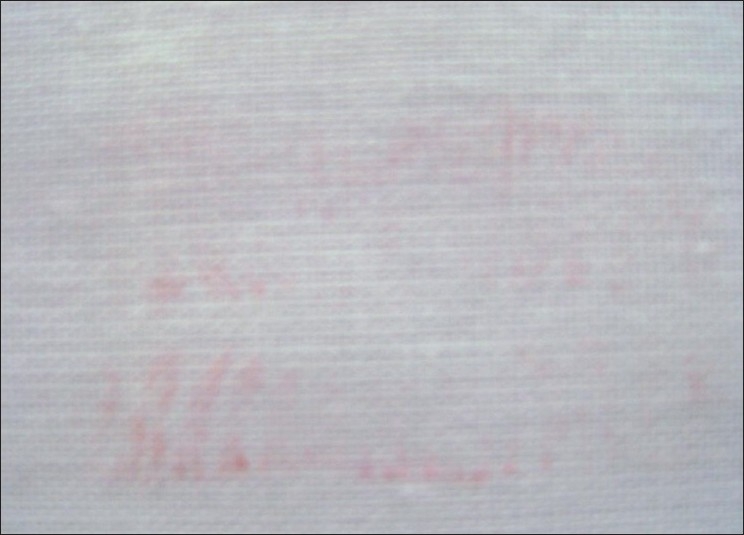
Latent lip prints

Latent Lip Prints [Figures 3 and 4]

**Figure 5:**
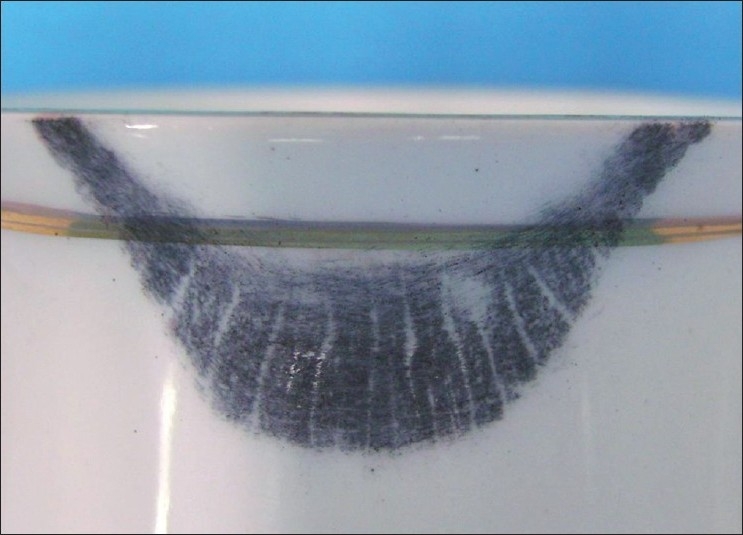
Visible lip print on cup - Sudan black

**Figure 6:**
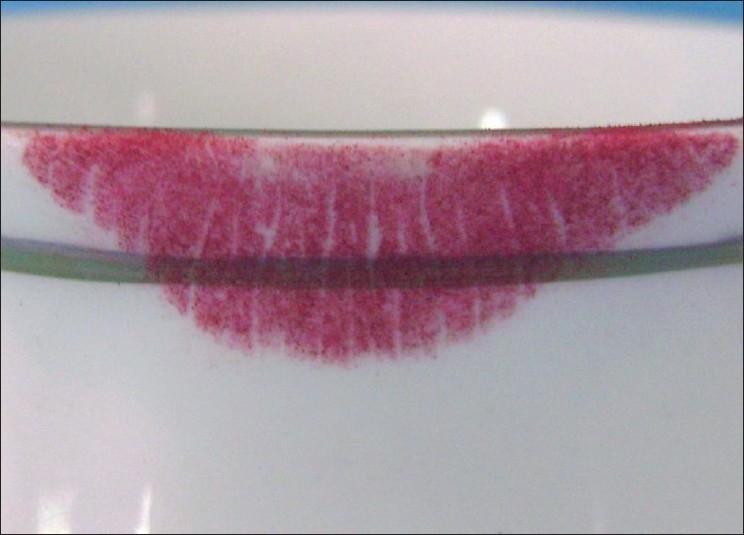
Visible lip print on cup - Vermilion

**Figure 7:**
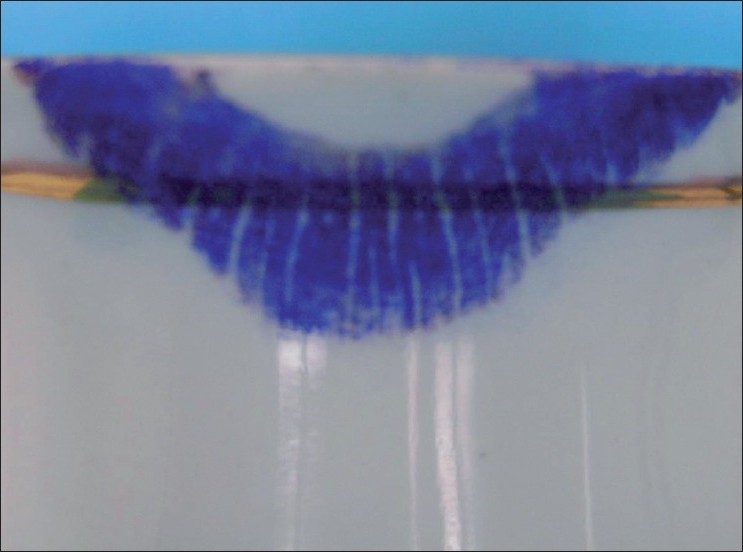
Visible lip print on cup - Indigo

Visible Lip Prints on Cup Developed with Sudan Black, Vermilion and Indigo [Figures 5–7]

**Figure 8:**
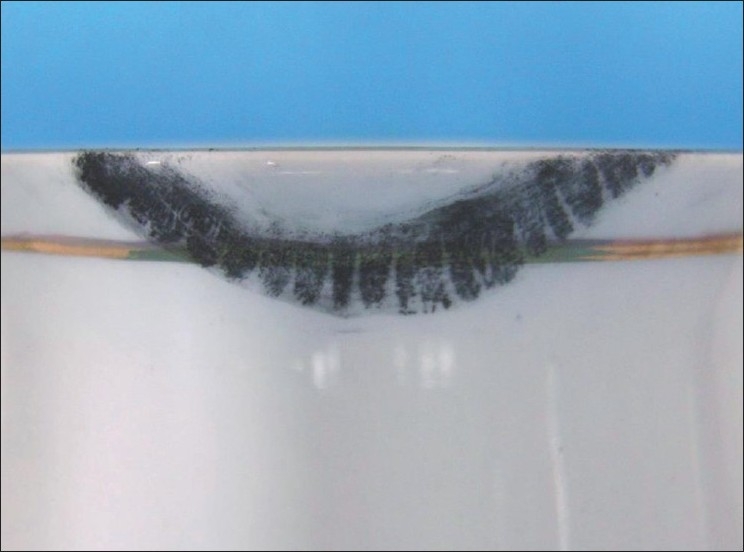
Latent lip print on cup - Sudan black

**Figure 9:**
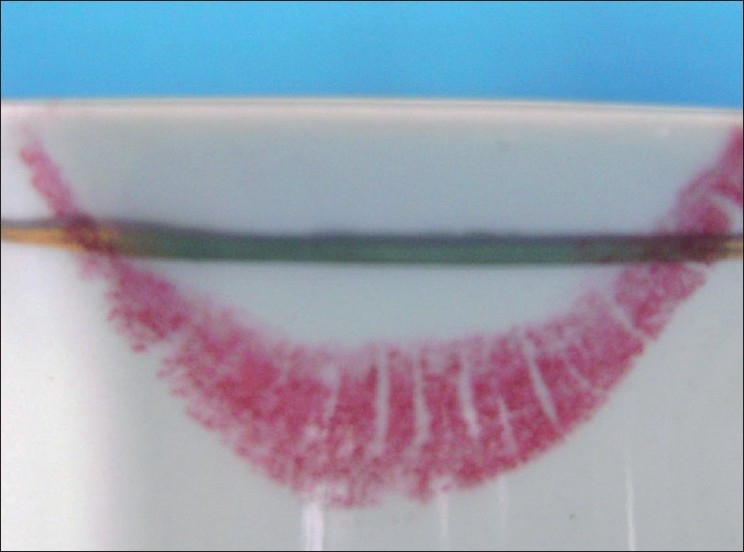
Latent lip print on cup - Vermilion

**Figure 10:**
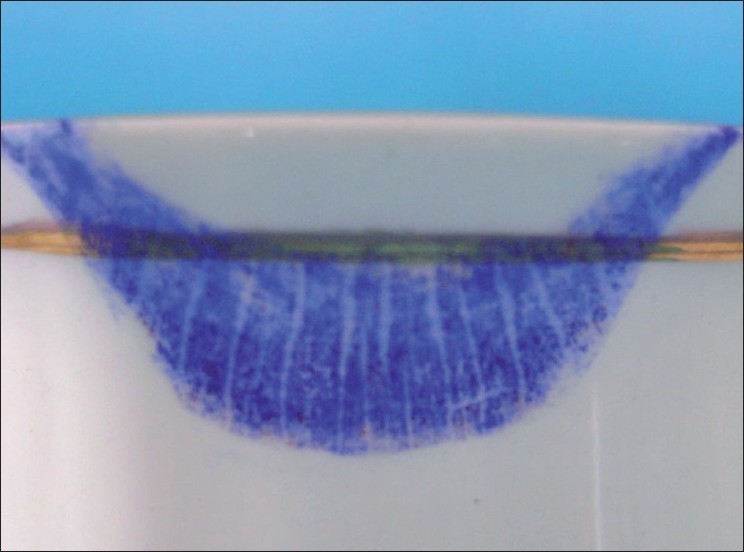
Latent lip print on cup - Indigo dye

Latent Lip Prints on Cup Developed with Sudan Black, Vermilion and Indigo Dye [Figures 8–10]

**Figure 11:**
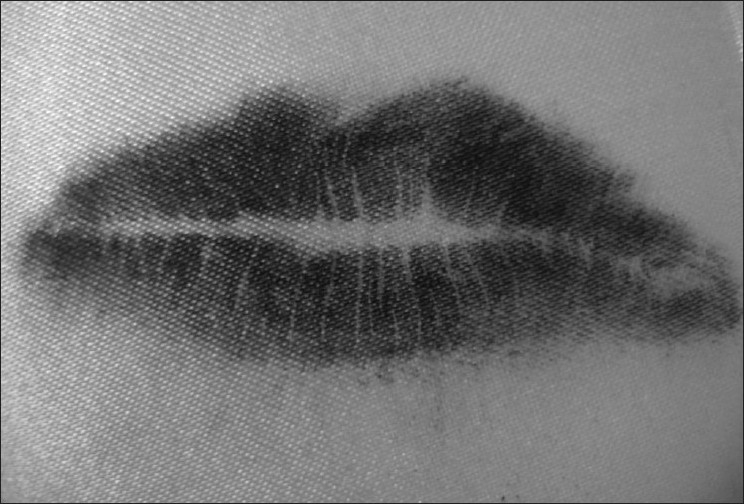
Visible lip print on satin fabric - Sudan black

**Figure 12:**
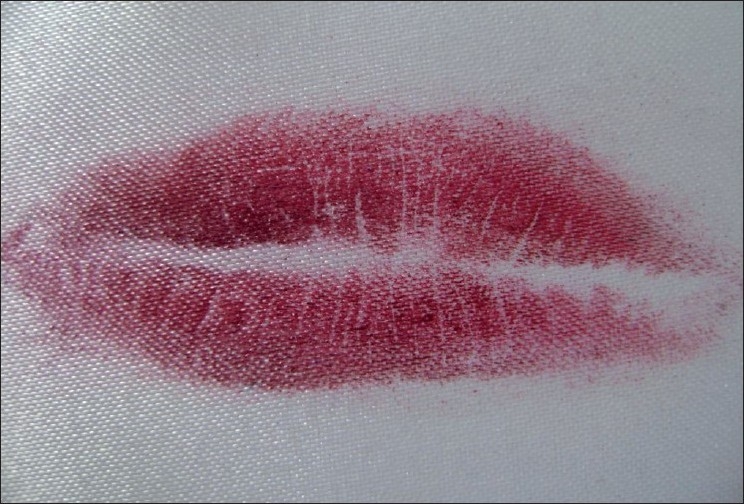
Visible lip print on satin fabric - Vermilion

**Figure 13:**
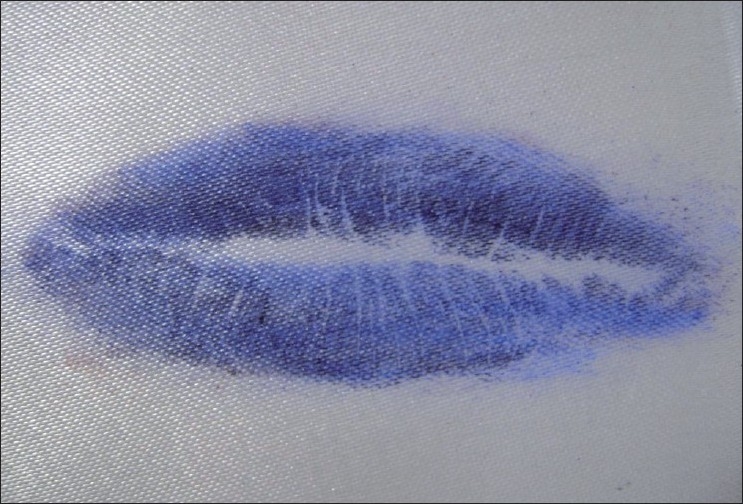
Visible lip print on satin fabric - Indigo dye

Visible Lip Prints on Satin Fabric Developed with Sudan Black, Vermilion and Indigo Dye [Figures 11–13]

**Figure 14:**
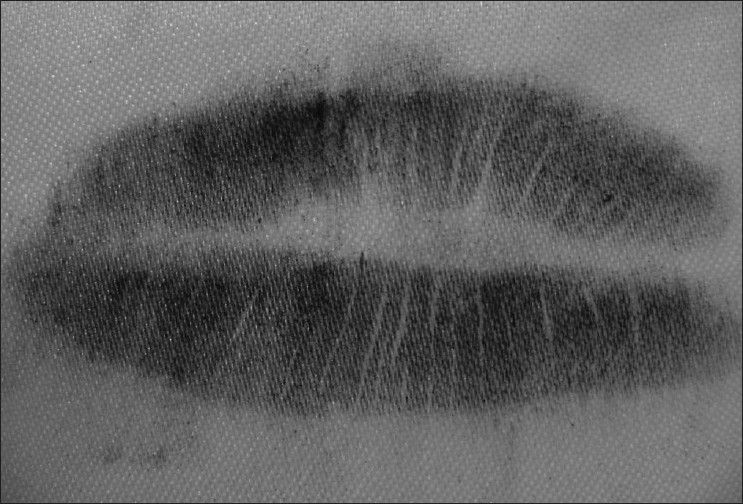
Latent lip print on satin fabric - Sudan black

**Figure 15:**
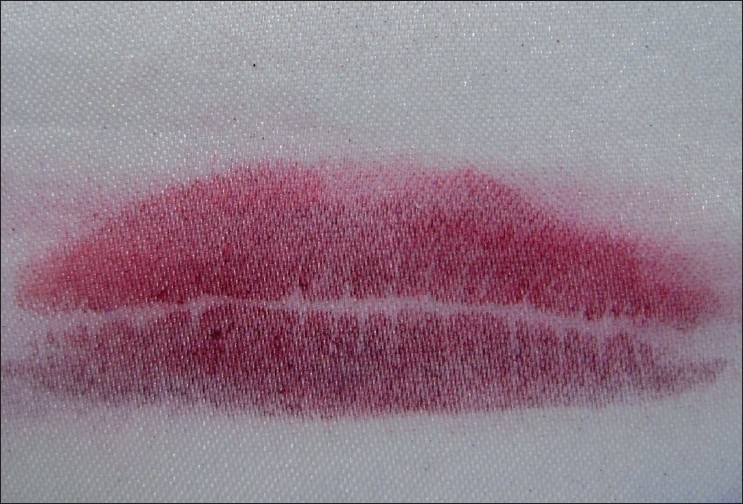
Latent lip print on satin fabric - Vermilion

**Figure 16:**
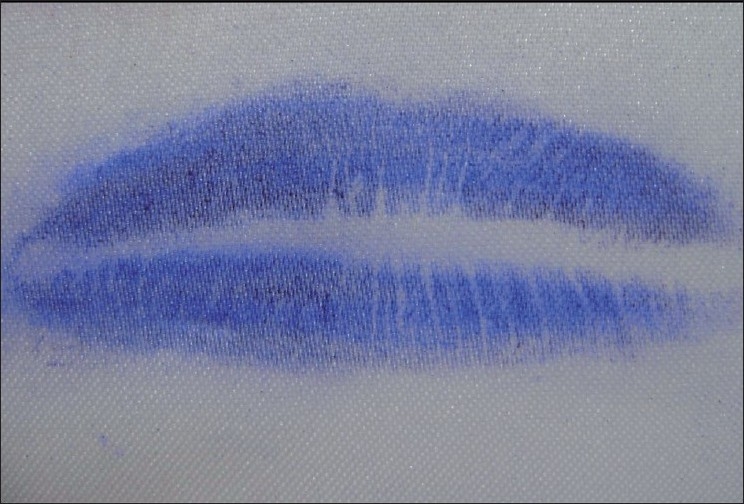
Latent lip print on satin fabric - Indigo dye

Latent Lip Prints on Satin Fabric Developed with Sudan Black, Vermilion and Indigo Dye [Figures 14–16]

**Figure 17:**
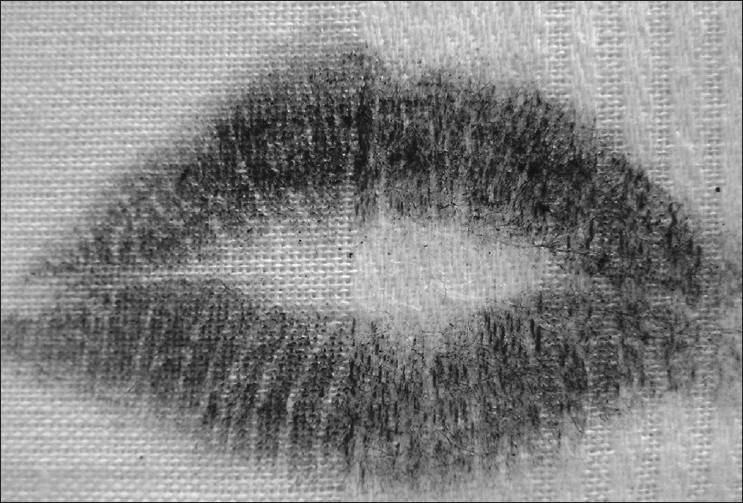
Visible lip print on cotton fabric - Sudan black

**Figure 18:**
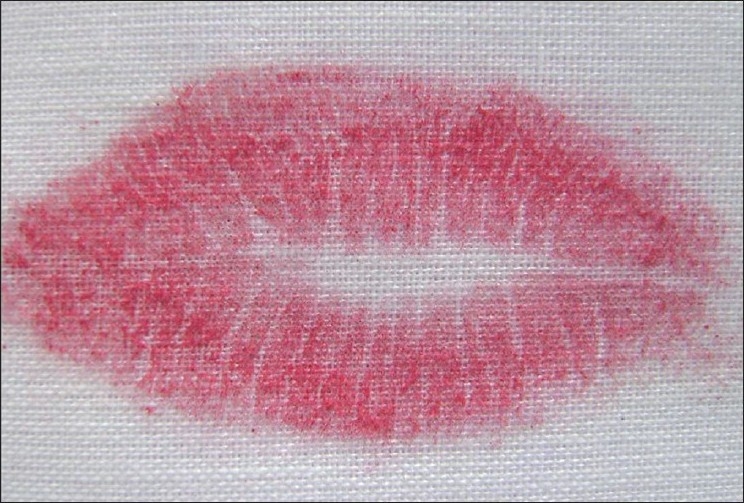
Visible lip print on cotton fabric - Vermilion

**Figure 19:**
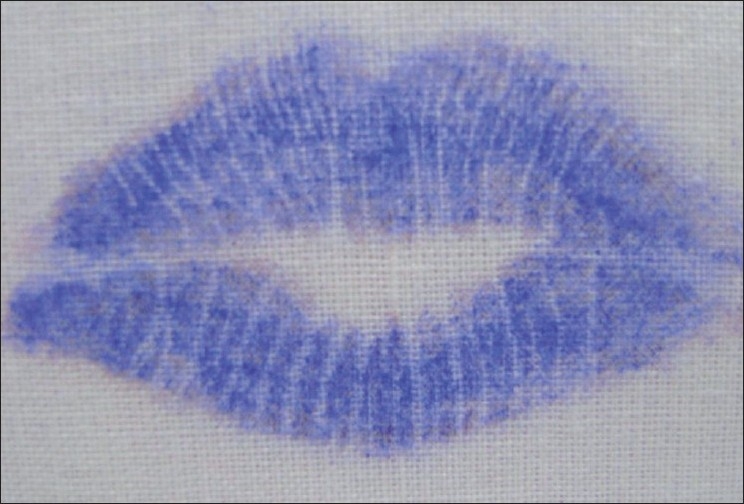
Visible lip print on cotton fabric - Indigo dye

Visible Lip Prints on Cotton Fabric Developed with Sudan Black, Vermilion and Indigo Dye [Figures 17–19]

**Figure 20:**
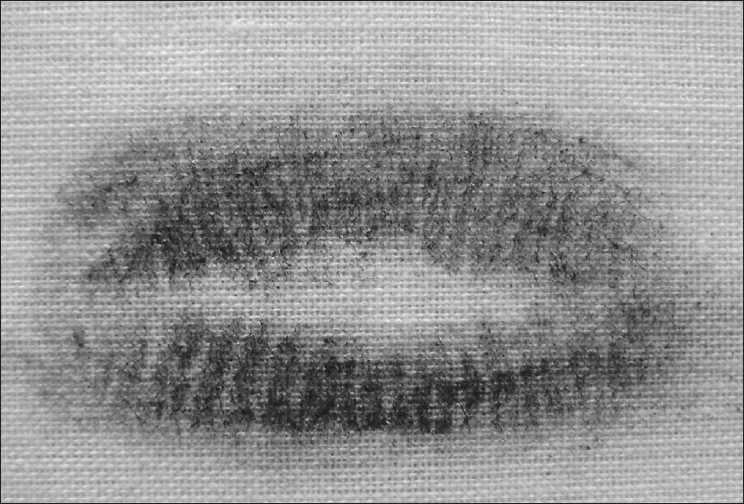
Latent lip print on cotton fabric - Sudan black

**Figure 21:**
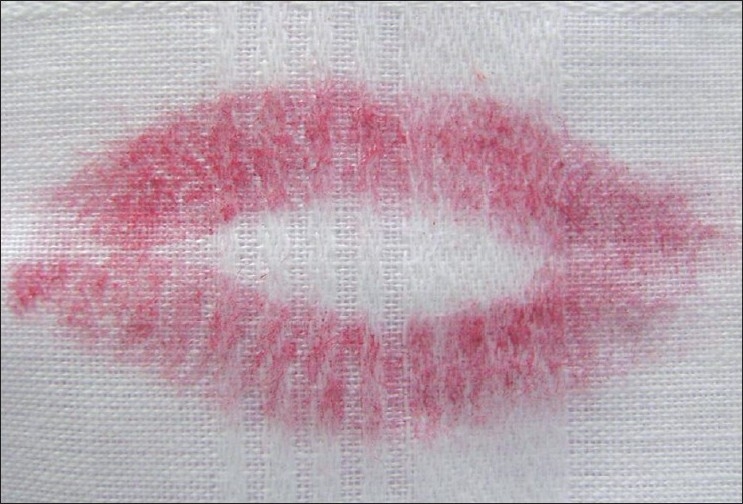
Latent lip print on cotton fabric - Vermilion

**Figure 22:**
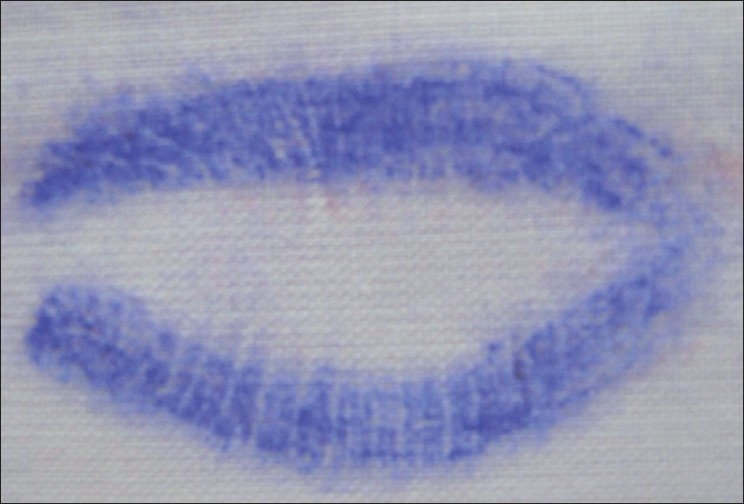
Latent lip print on cotton fabric - Indigo dye

Latent Lip Prints on Cotton Fabric Developed with Sudan Black, Vermilion and Indigo Dye [Figures 20–22]

The clarity of lip grooves developed depends on the type of reagent used and the surface on which lip prints were made. Visible and latent lip prints developed immediately with sudan black, vermilion, and indigo were compared using parameter of Good (++), Fair (+), and Poor (-) and statistically evaluated using the chi-square test keeping confidence limit at 95%.

GOOD (++): Lip outline and lip grooves that can easily be studied.

FAIR (+): Lip outline that can be noticed but with less clarity of lip grooves.

POOR (-): Lip outline can still be noticed but lip grooves cannot be appreciated.

**Figure 23:**
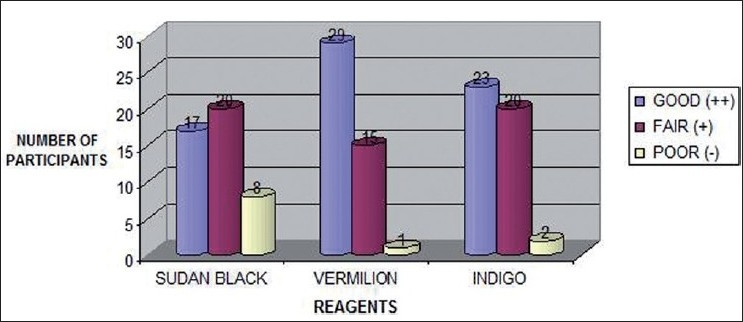
Reagent comparison among sudan black, vermilion and Indigo for lip print development

**Figure 24:**
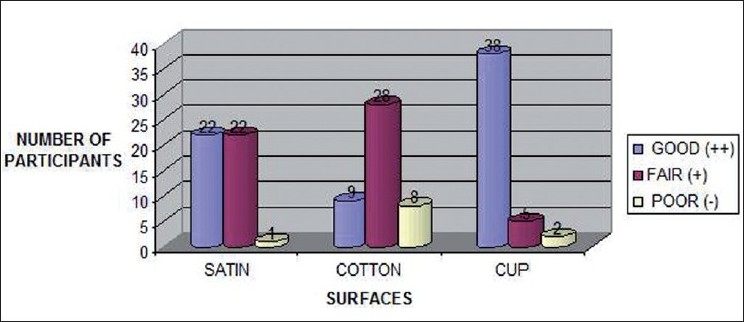
Surface comparison among satin, cotton and cup for lip print development

Results show [Figure 23, Tables 1 and 2] a statistically significant difference between sudan black and vermilion only (*P*=0.010). Though the proportion of lip prints developed with good results was higher with vermillion as compared to indigo and that of indigo as compared to sudan black, yet these differences were not found to be statistically significant. Cup was found to have significantly higher good lip prints developed as compared to both satin and cotton (*P*<0.001) [Figure 24, Tables 3 and 4], while satin was found to have significantly higher good lip prints developed as compared to cotton (*P*=0.003)

**Table 1:** Reagent comparison among sudan black, vermilion, and indigo for lip print development

	Good (++)	Fair (+)	Poor (-)
Sudan black	17	20	8
Vermilion	29	15	1
Indigo	23	20	2

**Table 2:** Statistical analysis for reagent comparison

Comparison	χ^2^	*P*
Sudan black vs. vermillion	9.289	0.010
Sudan black vs. Indigo	4.5	0.105
Vermilion vs. Indigo	1.740	0.419

**Table 3:** Surface comparison among satin, cotton, and cup for lip print development

	Good (++)	Fair (+)	Poor (-)
Satin	22	22	1
Cotton	9	28	8
Cup	38	5	2

**Table 4:** Statistical analysis for surface comparison

Comparison	χ^2^	*P*
Satin vs. cotton	11.616	0.003
Satin vs. cup	15.961	<0.001
Cotton vs. cup	37.524	<0.001

Attempts were made to develop latent prints obtained from non–lipstick-coated lips (as in males) with all the three dyes used, but none of them gave satisfactory results.

## Discussion

The quality development of lip print means that the lip outline and the lip grooves should be such, so that they could easily be studied and classified by the examiner. Also, the efficacy of developing lip prints by different chemical developers depends on the surface of the object on which lip print was made. Vermilion and indigo were better than sudan black, as they were free flowing and did not adhere to the surface of the object where lipstick smear was not present. The quality of lip prints developed was better on cup and satin fabric as compared to cotton fabric, probably due to greater absorbance of lipstick content by the cotton. Also the surface of cup and satin fabric is much more smooth and uniform when compared to cotton, hence better development. Visible lip prints are always better developed as compared to latent prints due to their high oil content so better they absorb the chemical reagent applied.

Vermilion (25 g for Rs.15.00) and indigo (50 g for Rs.12.00) are readily available and cost-effective chemical reagents in India, as compared to sudan black (25 g for Rs.1260.00), and the results have shown that both vermilion and indigo give comparable results to that of sudan black for development of recent lip prints, both visible and latent. However, according to the reported literature sudan black is an effective reagent for developing lip prints, since it is a lysochrome dye and all lip prints contain lipids.

Hence, the results of the aforementioned study signify that vermilion and indigo being natural, non-toxic, and cost-effective can replicate the already existing chemical reagents like sudan black, sudan III, oil red O, Nile red, as the ability of these natural dyes to develop recent lip prints are comparable to sudan black. However, further studies are required to ascertain the efficacy of these natural dyes to develop lip prints stored in variable conditions over a variable period of time.

Although, the use of natural dyes for studying lip prints is not inspired from the literature but the remarkable property of these dyes for development of lip prints can be a landmark in the field of forensic odontology and a pathway for further studies of this kind.
